# Improving citric acid production of an industrial *Aspergillus niger* CGMCC 10142: identification and overexpression of a high-affinity glucose transporter with different promoters

**DOI:** 10.1186/s12934-021-01659-3

**Published:** 2021-08-26

**Authors:** Xianli Xue, Futi Bi, Boya Liu, Jie Li, Lan Zhang, Jian Zhang, Qiang Gao, Depei Wang

**Affiliations:** 1 Key Laboratory of Industrial Microbiology & Engineering Research Center of Food Biotechnology of Ministry of Education, College of Biotechnology, Tianjin University of Science and Technology, Tianjin, 300457 People’s Republic of China; 2 Tianjin Key Laboratory of Industrial Fermentation Microbiology, Tianjin, 300457 People’s Republic of China; 3 Tianjin Engineering Research Center of Microbial Metabolism and Fermentation Process Control, Tianjin, 300457 People’s Republic of China

**Keywords:** *Aspergillus niger*, Glucose transporter, HGT1, Citric acid fermentation

## Abstract

**Background:**

Glucose transporters play an important role in the fermentation of citric acid. In this study, a high-affinity glucose transporter (HGT1) was identified and overexpressed in the industrial strain *A. niger* CGMCC 10142. HGT1-overexpressing strains using the P*gla*A and P*aox1* promoters were constructed to verify the glucose transporter functions.

**Result:**

As hypothesized, the HGT1-overexpressing strains showed higher citric acid production and lower residual sugar contents. The best-performing strain *A. niger* 20-15 exhibited a reduction of the total sugar content and residual reducing sugars by 16.5 and 44.7%, while the final citric acid production was significantly increased to 174.1 g/L, representing a 7.3% increase compared to *A. niger* CGMCC 10142. Measurement of the mRNA expression levels of relevant genes at different time-points during the fermentation indicated that in addition to HGT1, citrate synthase and glucokinase were also expressed at higher levels in the overexpression strains.

**Conclusion:**

The results indicate that HGT1 overexpression resolved the metabolic bottleneck caused by insufficient sugar transport and thereby improved the sugar utilization rate. This study demonstrates the usefulness of the high-affinity glucose transporter HGT1 for improving the citric acid fermentation process of *Aspergillus niger* CGMCC 10142.

**Supplementary Information:**

The online version contains supplementary material available at 10.1186/s12934-021-01659-3.

## Background

Citric acid (CA) is a naturally occurring tricarboxylic acid that is widely used in food, chemistry, medicine, and other fields, whose high demand places it among the most commonly used organic acids in the world [1, 2]. Moreover, the demand for microbially fermented CA has been increasing at an annual rate of 3.7% in recent years. Nowadays, CA is mostly produced using *Aspergillus niger* fermentation on the industrial scale. As a saprophytic fungus, *A. niger* secretes abundant hydrolytic enzymes which are beneficial for CA production on complex substrates [3, 4].

*A. niger* is one of the most important industrial microorganisms, with tremendous commercial value. With the development of sequencing and genomic technologies, rational molecular evolution and modification instead of random mutagenesis are increasingly being applied to increase the CA fermentation efficiency of *A. niger* [5]. To date, the principal metabolic engineering strategies in the CA industry focus on the improvement of the central metabolic fluxes and the efficiency of energy generation in the respiratory chain of *A. niger* [6–8], mostly via knockout and overexpression of key genes involved in the EMP (glycolysis) pathway, the TCA cycle, and electron transport [9–11]. Recent studies focused on energy metabolism and electron transport [12, 13]. The metabolism of *A. niger* includes a complex network of replenishment pathways, which can overcome the effects of individual gene overexpression, leading to limited improvement of citric acid production. For example, the overexpression or deletion of citrate synthase did not improve citric acid production [14, 15]. Thus, this enzyme does not catalyze a rate-limiting step.

Accordingly, Torres et al. calculated that the key limiting points of citric acid production are the intake and phosphorylation of hexoses [16]. If a high glucose concentration is maintained during citric acid fermentation, the CA production peak period of the fermentation process will be advanced, but the final yield will not increase [17]. In general terms, the type of carbon source and its concentration are both critical factors determining the efficiency of citric acid fermentation. Xu et al. found that adding 10% w/v of sugars, such as maltose, sucrose, mannose or fructose, led to the highest yields of citric acid, while 75 g/L glucose concentration gave the best results. Furthermore, there was no citric acid production in the media with less than 2.5% sugar [18]. Mischak et al. clearly showed that the uptake of glucose was inhibited by small quantities of extracellular citric acid, at concentrations over 0.5 mM [19]. Thus, citric acid production and its rate are strongly related to glucose concentration and its uptake rate. Furthermore, the absorption rate of glucose exhibits a simple linear relationship with the glucose concentration, and as a highly hydrophilic molecule, glucose cannot passively pass through the cell membrane. Accordingly, facilitated diffusion and active transporter are the main glucose uptake modes [20].

There are two types of glucose transporters in *A. niger*, a low-affinity glucose transporter with *K*_m_ of 3.67 mmol/L, and a high-affinity glucose transporter with *K*_m_ of 260 µmol/L. Torres et al. found that a high glucose concentration (> 50 g/L) is needed for the low-affinity glucose transporter to become active, which is capable of providing the high flux of glucose required for citrate production [16]. Transcriptomic analysis of *A. niger* H915-1 during the fermentation process revealed that a low-affinity glucose transporter maintained high transcript levels, while 5 high-affinity glucose transporters did not [20]. This native state of *A. niger* with a low-affinity glucose transporter with high *K*_m_ and high-affinity glucose transporters with extremely low-expressed levels, can explain the limited citrate yields and large amounts of residual glucose in the fermentation broth [21, 22]. Controlling and coordinating these proteins can change the nutrient uptake ability of the strains in a targeted manner, making them more suitable for industrial fermentation. Therefore, the glucose transport efficiency of *A. niger* is crucial for increasing CA production and may be one of the limiting factors for obtaining higher CA yields [21, 22].

Many putative sugar transporters have been identified in fungi. Interestingly, several newly discovered xylose transporters from *A. nidulans*, *A. niger*, and *T. reesei* can also transport glucose [23, 24]. Additionally, the low-affinity glucose transporter HxtB, which is involved in glucose signaling was also characterized [25]. However, there are a few reports on the specific glucose transporters of *A. niger*. Based on a conserved protein domain search, 86 putative sugar transporter genes were identified and annotated in the genome of *A. niger* [26]. Two new putative high-affinity glucose transporters, named MstG and MstH, were identified in *A. niger* by membrane‑associated proteome analysis and biochemically characterized [27]. Additionally, it was found that MstA is a high-affinity glucose transporter in *A. niger*, and its disruption resulted in a two- to fivefold reduction of cellular glucose uptake [28]. This protein is expressed by the enzyme-producing strain *A. niger* CBS 513.88 as well as the original industrial acid-producing strain *A. niger* ATCC 1015 [29], but it is not expressed by *A. niger* CGMCC 10142. Additionally, two glucose transporters were identified in *A. niger* H915-1, a low-affinity glucose transporter with high expression at high glucose concentration, and a high-affinity glucose transporter with extremely low-expression level [20]. Liu et al. increased the glucose transport rate and shortened the fermentation cycle by overexpressing a low-affinity glucose transporter [30]. Notably, the glucose transporter HGT1 (high-affinity glucose transporter 1), first identified in *Kluyveromyces lactis*, was also found in the *A. niger* CBS 513.88 and *A. niger* CGMCC 10142 [31, 32].

The primary substrate used in the *A. niger* CA industry is corn steep liquor with high amounts of total sugars, including glucose, disaccharides, and polysaccharides. *A. niger* secretes abundant glucoamylases that can hydrolyze polysaccharides into readily bioavailable glucose. Thus, the main carbon source at the beginning of the fermentation is glucose, and an adequate supply of carbon sources can greatly increase the CA production. Undoubtedly, the transport rate of glucose understandably becomes a limiting factor preventing the further increase of CA yields. Therefore, it is desirable to accelerate the transport of glucose during the fermentation of *A. niger* and thereby improve the citric acid production performance.

Here, the industrial CA-production strain *A. niger* CGMCC 10142 was used as the starting strain for metabolic engineering. The gene encoding the high-affinity glucose transporter HGT1 was identified and cloned from the parental strain. After sequence alignment, the strong endogenous P*gla*A and P*aox1* promoters were selected to overexpress HGT1. Subsequently, HGT1-overexpressing strains were successfully constructed and characterized. The transcription levels of key enzyme genes involved in CA production were analyzed during the fermentation by real-time qPCR. Furthermore, the biomass and residual sugars were quantified and compared between the engineered strains and the parental strain.

## Results and discussion

### Sequence analysis of the native HGT1 from the parental strain

**Fig. 1:**
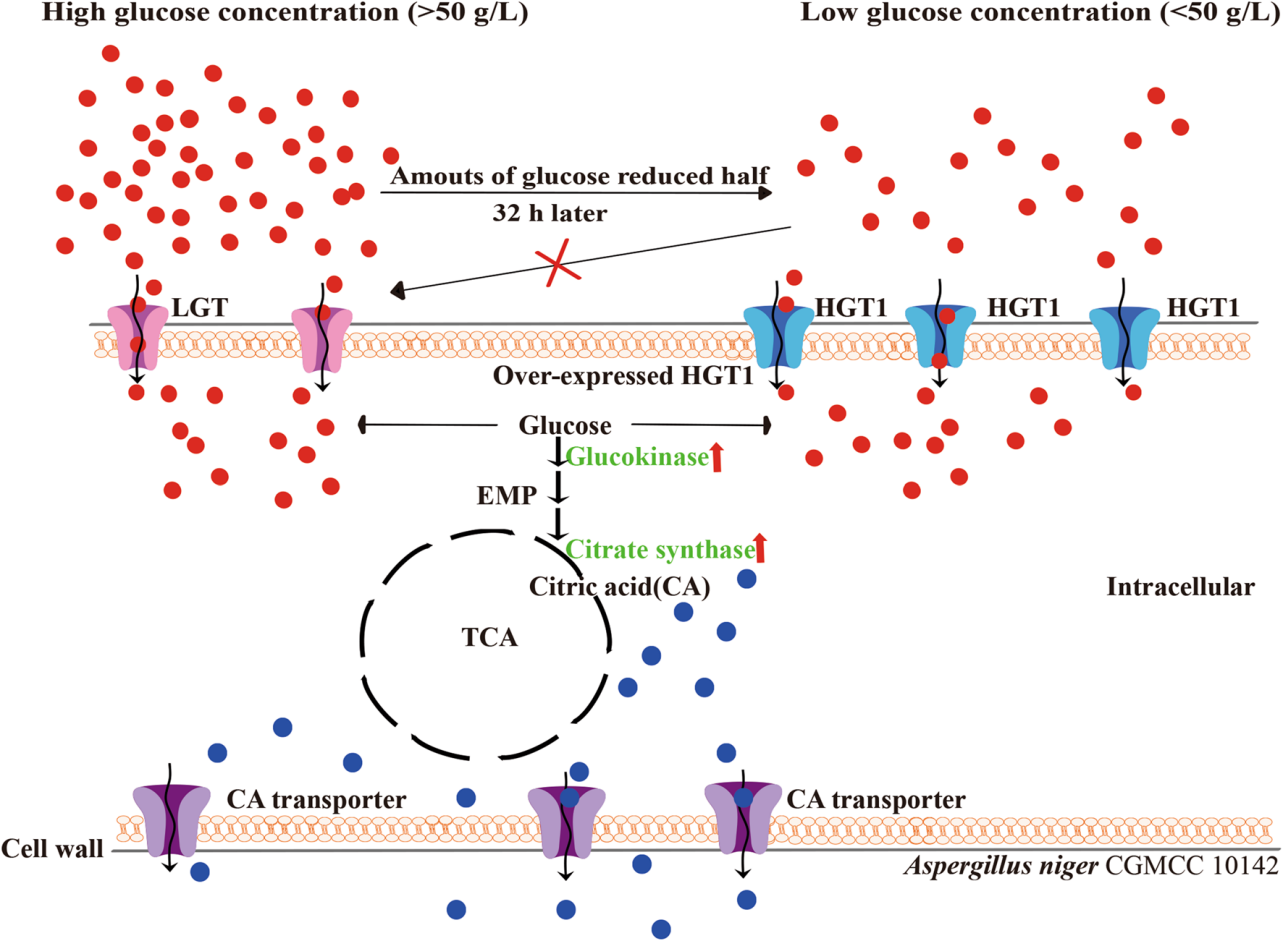
The schemes of the passway on glucose-citric acid conversion including that how LGT and HGT1 transport glucose

The low-affinity glucose transporter is the limiting factor for glucose import into the cell in fermentation at relatively higher glucose concentrations (> 50 g/L) [20]. By contrast, the high-affinity glucose transporters can function even at the end of fermentation when the sugar concentration is extremely low, as depicted in Fig. 1.

The genome of the industrial CA production strain *A. niger* CGMCC 10142 was re-sequenced by our team and further analyzed to screen essential high-affinity glucose transporters. The high-affinity glucose transporter HGT1 was identified at the locus contig1 (6,084,010:6,085,828) and a BLASTn showed that it was 100% identical to its ortholog from *A. niger* CBS 513.88 (GenBank: AM269996.1). It is remarkable that the high-affinity glucose transporters show practically no-transcription at 12 h of fermentation. To improve the glucose utilization rate, the two plasmids p20 and p21 for the overexpression of HGT1 using the promoters P*gla*A and P*aox1* were successfully constructed (Figures S1a and S1b). Both plasmids were verified by PCR and sequenced as shown in Figures S2a and S2b.

The amino acid sequence of *A. niger* HGT1 (XP_001391024.1) was aligned with those of MstA (AAL89822.1) [28], LGT1 (XP_001399490.1) [30], and *A. nidulans* MstE (XP_663464.1) [25]. The respective similarity of the amino acid sequences was 27.83, 25.71, 25.91%, respectively (Additional file 1: Figure S3). The amino acid sequence identity between HGT1 from *A. niger* and its homolog (AAC49461.1) from *Kluyveromyces lactis* reached 40.87% [31]. In this study, the structure of HGT1 from *A. niger* was predicted through homology modeling in Swiss-model using the sugar transport protein STP10 (PDB:6H7D_A) [33] and D-xylose-proton symporter GLUT1–4 (PDB: 4GBY_A) [34] as the template.

**Fig. 2:**
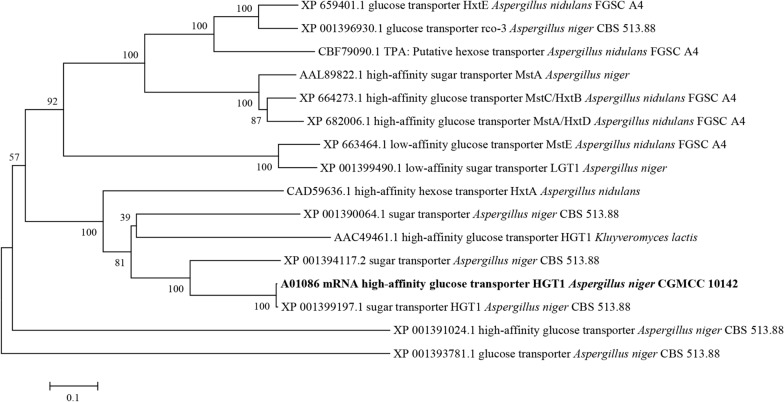
Phylogenetic tree of HGT1 from *Aspergillus niger* CGMCC 10142 (A01086) with other homologs. Phylogenetic tree of HGT1 and other homologs from *Aspergillus nidulans* FGSC A4 (XP 659401.1), *Aspergillus niger* CBS 513.88 (XP 001396930.1), *Aspergillus nidulans* FGSC A4 (CBF79090.1), *Aspergillus nidulans* FGSC A4 (CBF79090.1), *Aspergillus niger* (AAL89822.1), *Aspergillus nidulans* FGSC A4 (XP 664273.1), *Aspergillus nidulans* FGSC A4 (XP 682006.1), *Aspergillus nidulans* FGSC A4 (XP 663464.1), *Aspergillus niger* (XP 001399490.1), *Aspergillus nidulans* (CAD59636.1), *Aspergillus niger* CBS 513.88 (XP 001390064.1), *Kluyveromyces lactis* (AAC49461.1), *Aspergillus niger* CBS 513.88 (XP 001394117.2), *Aspergillus niger* CBS 513.88 (XP 001399197.1), *Aspergillus niger* CBS 513.88 (XP 001391024.1), *Aspergillus niger* CBS 513.88 (XP 001393781.1). The tree was established via Neighbor-joining (NJ) method in MEGA 5.05 version. The scale bar corresponds to 0.1 estimated amino acid substitutions per site;

A phylogenetic tree of sugar (glucose) transporters from different fungi is shown in Fig. 2. Several glucose transporters have been identified in both *A. niger* and *A. nidulans.* The HGT1 of *A. niger* CGMCC 10142 corresponds to CBS 513.88 HGT1 (XP_001399197.1) of *A.niger*. Several other glucose transporters that are close to HGT1 included XP_001394117.2 and XP_001390064.1. What’s more, the MstA in *A. niger* and most of the sugar transporters identified in *A. nidulans* were located in the same branch of the phylogenetic tree. Additionally, several different sugar transporters were identified in *A. nidulans* and *A. niger*, which was consistent with previous studies. Several different high-affinity sugar transporters are encoded in the genome of *A. niger*, and they may not be closely related according to the phylogenetic analysis (collapsing branches at an average branch length distance < 0.6). It is possible that sugar assimilation constraints under different environmental conditions led to the evolution of a diverse array of glucose transporters, whereby their transcriptional regulation and ecological role remain mostly unclear.

### Verification of expression cassettes integration and genetic stability of the transformants

Firstly, The P*gla*A-*HGT1*, P*aox1-HGT1*, and *HGT1-hyg* fragments of the transformants were amplified to verify successful cloning of the c*assette into A. tumefaciens* (Additional file 1: Figure S2c), and subsequently inserted into the genomic DNA of *A. niger* (Figures S2d, S2e, and S2f). Then, the integration location was determined using primers spanning the total length of *ku70*. If the plasmid was homologously recombined to the *ku70* gene, a 5710 bp fragment including the homologous bodies of *ku70* and the *HGT1*-overexpression cassette could only be amplified by PCR, while the *HGT1*-overexpression cassette was randomly integrated into the genome, only an 1800 bp fragment of *ku70* gene itself can be amplified. As shown in Additional file 1: Figure S2g, the original length of *ku70* (1800 bp), and not the target fragment (5710 bp) was amplified from the genomic DNA of *A. niger*. Thus, the results confirmed that the whole P*gla*A/P*aox1*-*HGT1*-*hyg* segment was randomly inserted into the genome of the transformants. Furthermore, the 8 genetically modified strains were passaged for 15 generations and transformants’ genetic stability was confirmed by validating the *hyg* gene sequence as shown in Additional file 1: Figure S4. Then, conidia were prepared from the 8 identified stable transformants to verify their CA production ability in shake-flask fermentation.

### Screening of strains for higher CA production in shake-flask fermentation

**Fig. 3:**
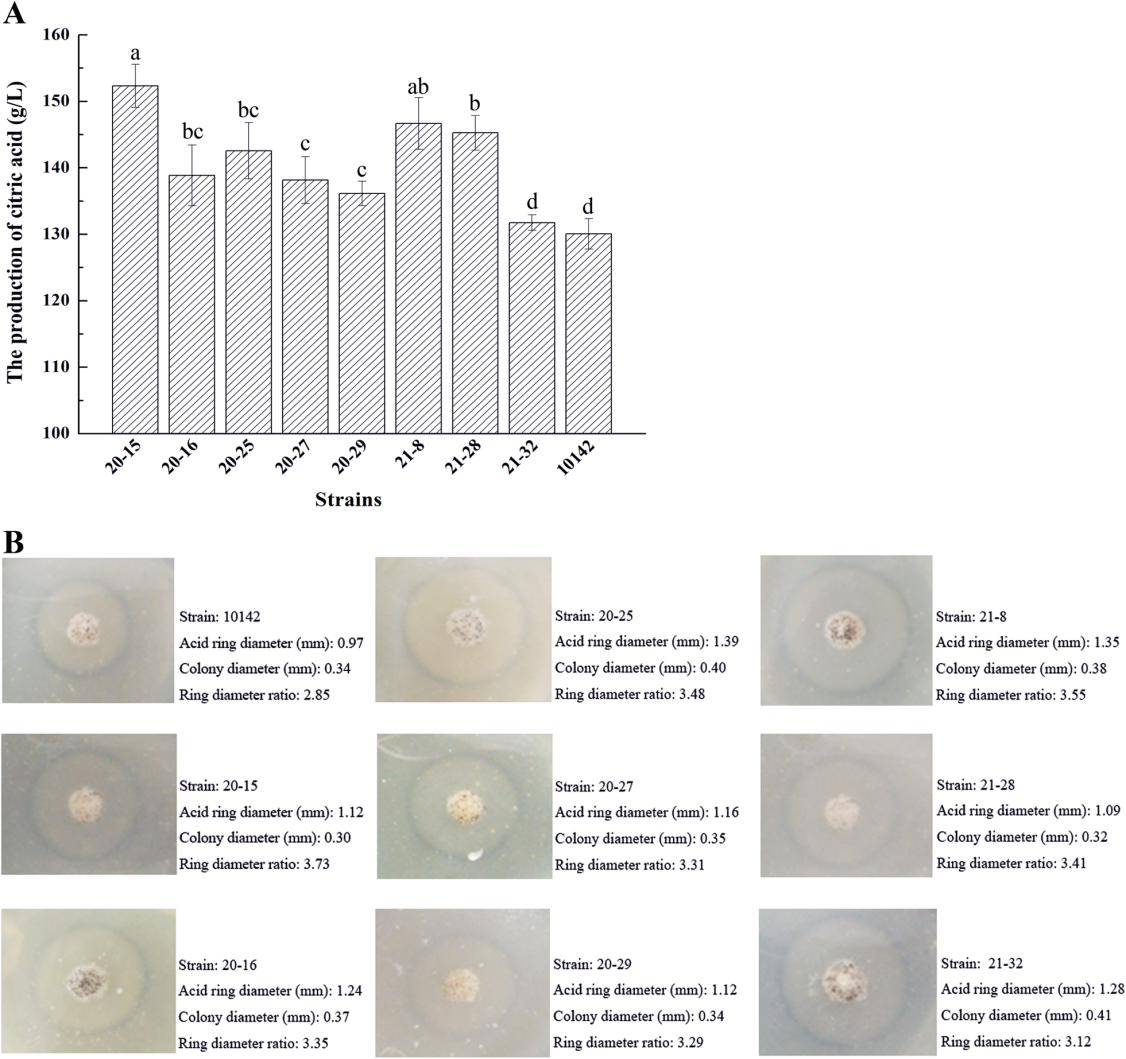
Verification of citric acid production of transformants by the shake flask fermentation and Ring diameter ratios. **A** Verification of citric acid production of transformants (20-15, 20-16, 20-25, 20-27, 20-29, 21-8, 21-28 and 21-32) by the 50 mL shake flask fermentation; **B** Ring diameter ratios determination of transformants (20-15, 20-16, 20-25, 20-27, 20-29, 21-8, 21-28 and 21-32) by transparent halo zone on CM + CaCO_3_ medium. CA yield with different letters differ significantly (abcd, P < 0.05)

Initially, 68 positive transformants were screened by calculating the ratios of the transparent halo zones due to acid production and colony diameter (Additional file 2: Table S1). Eight superior transformants (*A. niger* 20-15, 20-16, 20-25, 20-27, 20-29, 21-8, 21-28, and 21-32) were selected for shake flask fermentation to screen higher yielding transformants (Fig. 3A). Comparing to the 130.1 g/L CA titer of *A.niger* CGMCC 10142, the CA production of seven transformants was increased, and among them transformants 20-15, 20-25, 21-8 and 21-28 exhibited significant improvements of CA production to 152.3 g/L (+ 17.1%), 144.6 g/L (+ 11.1%), 146.7 g/L (+ 12.8%) and 145.3 g/L (+ 11.7%), respectively (Fig. 3A). This was consistent with the ratios of the transparent halo zones of 20-15 with 3.73, 20-25 with 3.48, 21-8 with 3.55, and 21-28 with 3.41, compared to *A. niger* CGMCC 10142 with a ratio of 2.85 (Fig. 3B). Subsequently, the transformants 20-15 and 21-8 were selected for further experiments. The growth speed of transformants 20-15 and 21-8 were faster than the parental strain under low glucose (less than 1%) fungal complete medium (CM) [35] plate could confirm that HGT1 could facilitate the uptake of glucose thus accelerating the growth of the strain.

### CA fermentation in bioreactor-scale fermentations and statistical analysis

**Fig. 4:**
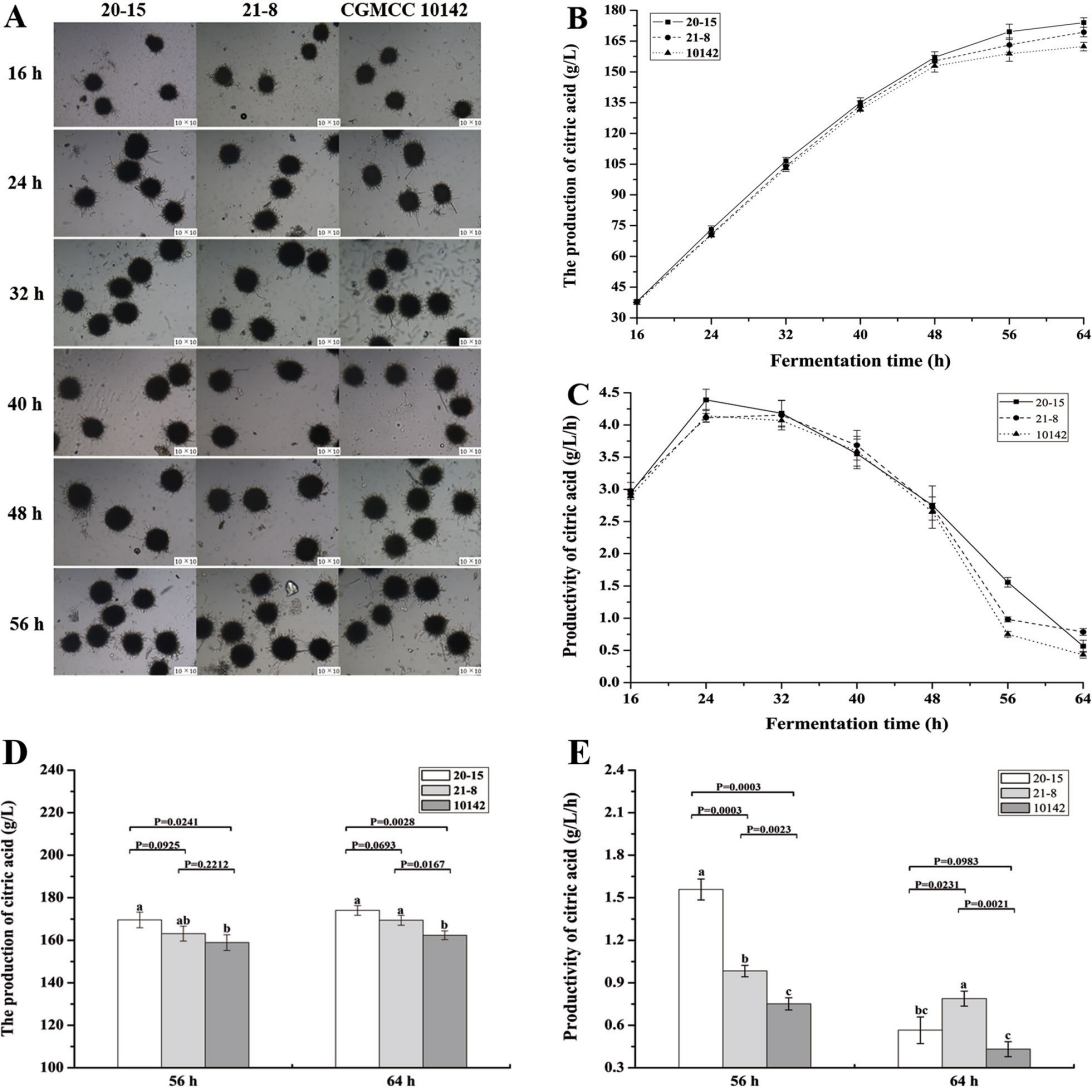
Observation of mycelial morphology and verification of production and productivity of citric acid in the 30 L bioreactor fermentation. **A** Observation of mycelial morphology of *A. niger* 20-15 (20-15), *A. niger* 21-8 (21-8) and *A. niger* CGMCC 10142 (10142) strain at different time points of fermentation; **B** Verification of citric acid production of 20-15, 21-8 and 10142 strain at different time points in the 30 L bioreactor fermentation; **C** Verification of citric acid productivity of 20-15, 21-8 and 10142 strain at different time points in the 30 L bioreactor fermentation; **D** The citric acid production of 20-15, 21-8 and 10142 strain at 56 and 64 h in the 30 L bioreactor fermentation;** E** The citric acid productivity of 20-15, 21-8 and 10142 strain at 56 and 64 h in the 30 L bioreactor fermentation. The samples at each time-point with different letters differ significantly (abc, P < 0.05)

The morphology of mycelial pellets at different times during CA fermentation is shown in Fig. 4A. The morphology of the parental *A. niger* strain CGMCC 10142, as well as the transformants *A. niger* 20-15 and 21-8, was normal at each sampling time. The diameter of the mycelial pellets increased quickly before 24 h, showing that the strains had entered the rapid growth phase. After 24 h the pellets of fungi showed a little increase and the colony began to sprout small mycelium, indicating that has begun to enter the period of CA production. Papagianni et al. observed a reduction of mycelial clumps for the first 48 h of fermentation [36], but in our research, the colony morphology did not change significantly from the period of CA production until the end of fermentation. Furthermore, the mycelial pellets of *A. niger* 21-8 still showed some growth from 24 to 32 h and maintained morphological stability until the end. After 32 h, all strains had similar mycelial pellet size and remained stable as shown in Fig. 4A and Additional file 1: Figure S5. Since no new spores were produced in the liquid fermentation, all strains had similar biomass after 32 h of fermentation.

The CA production in the bioreactor-scale fermentation was also measured every 8 h, as shown in Figs. 4B and D. The CA production increased rapidly from 16 h, and the transformants showed a gradually improved CA fermentation ability. The CA production of *A. niger* 20-15 and 21-8 increased to 174.1 g/L and 169.4 g/L, representing respective 7.3% (P < 0.01) and 4.4% (P < 0.05) improvements compared to the 162.3 g/L CA titer of strain 10142. The difference between *A. niger* 20-15 and 21-8 was not statistically significant (P > 0.05). The productivity at each fermentation stage was calculated, as shown in Figs. 4C and E. The highest production rate of *A. niger* 20-15 reached 4.4 g/L/h, which was higher than both *A. niger* 21-8 and 10142, which respectively reached 4.1 g/L/h at 24 h of fermentation. The highest productivity of *A. niger* 21-8 reached 4.2 g/L/h, which was approximately equal to *A. niger* 20-15 but higher than *A. niger* CGMCC 10142 with 4.0 g/L/h at 32 h, and remained at a high level at 40 h. This indicated that CA production at 24 h was higher in *A. niger* 20-15 than in *A. niger* 21-8 or *A. niger* CGMCC 10142, probably due to the fact that 21-8 did not have such a strong HGT1 overexpression promoter. The biggest differences in productivity were observed between 56 to 64 h at the end of fermentation. During this time, the CA productivity of *A. niger* 20-15 and *A. niger* 21-8 was 1.6-0.6 g/L/h and 1.0-0.7 g/L/h, respectively, which was 2.1-1.5 and 1.3-1.8 times higher than that of *A. niger* CGMCC 10142, which reached 0.7-0.4 g/L/h. There was a significant difference among the three strains (P < 0.01) at 56 h, and there was also a significant difference between *A. niger* 21-8 and *A. niger* CGMCC 10142 (P < 0.01) or *A. niger* 20-15 (P < 0.05). However, there was no significant difference between *A. niger* 20-15 and *A. niger* CGMCC 10142 (P > 0.05) at 64 h. These results further confirmed that the overexpression of HGT1 promoted CA production at the later stage of fermentation when the carbon source was nearing exhaustion.

**Fig. 5:**
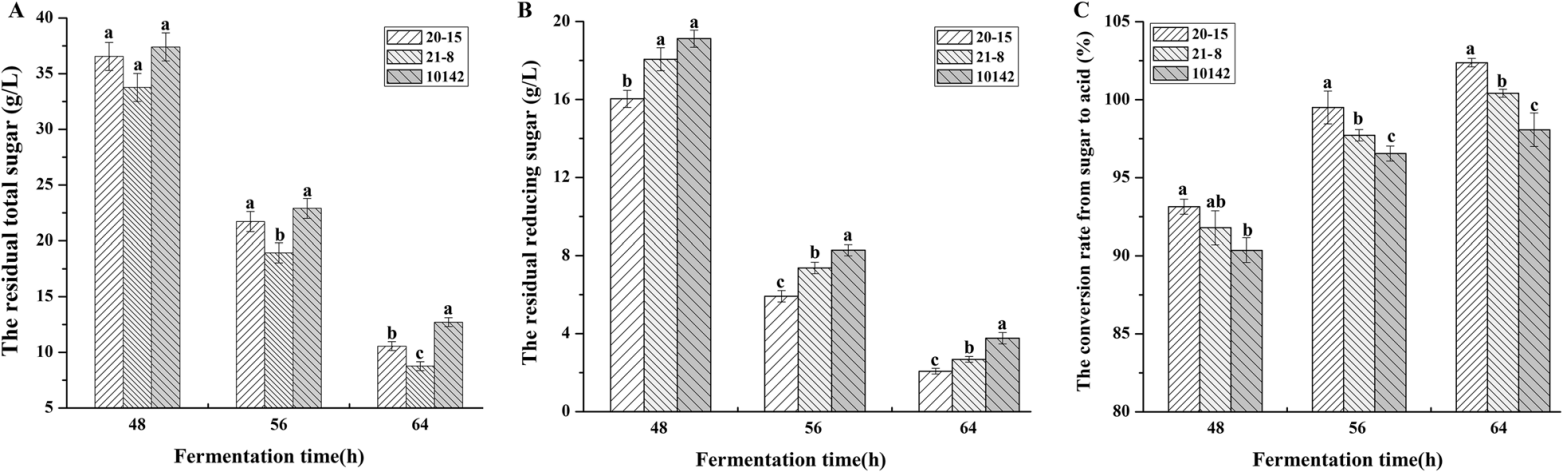
The CA fermentation performance in the 30 L bioreactor. **A** The residual total sugar changes in the fermentation broth at 48, 56, and 64 h; **B** The residual reducing sugar changes in the fermentation broth at 48, 56, and 64 h; **C** The conversion rate from sugar to CA in the fermentation broth at 48, 56, and 64 h. The samples at each time-point with different letters differ significantly (abc, P < 0.05)

During the fermentation process, the total sugar in the fermentation broth was measured every 8 h. The concentrations of residual total sugar at 48, 56, and 64 h were 36.6, 21.7, and 10.6 g/L for *A. niger* 20-15, which were higher than the corresponding values for *A. niger* 21-8, at 33.8, 18.9, and 8.8 g/L, but lower than those of *A. niger* CGMCC 10142, at 37.4, 22.9, and 12.7 g/L, respectively (Fig. 5A). This indicated that the overexpression of HGT1 had a significant positive effect at 56 and 64 h of the fermentation. These results were consistent with an earlier study [32]. Earlier studies found that low-affinity glucose transporters are expressed when the glucose concentration reaches 15%, and are highly expressed when it drops to 8% [20, 21]. Our results also showed that the most significant changes were observed when the sugar concentration dropped below 8% at 32 h, whereby HGT1 began to play a more important role. At the end of fermentation (64 h), the total residual sugar of *A. niger* 20-15 and *A. niger* 21-8 decreased 16.5 and 30.7% compared with *A. niger* CGMCC 10142, respectively. This result indicated that the overexpression of HGT1 significantly increased the sugar consumption rate.

A proportion of the initial sugar was not utilized by the microorganisms during CA fermentation, leading to waste of raw materials. Therefore, the total reducing sugar concentrations in the fermentation broth were measured at 48, 56, and 64 h as shown in Fig. 5B. The total reducing sugar concentrations at 48, 56, and 64 h were 16.0, 5.9, and 2.1 g/L of *A. niger* 20-15, which were less than the corresponding values of *A. niger* 21-8, at 18.1, 7.4, and 2.7 g/L, respectively. Both genetically modified strains exhibited lower residual sugar concentrations than the parental strain *A. niger* CGMCC 10142, which left 19.1, 8.3, and 3.8 g/L of sugar utilized. Accordingly, the final total reducing sugar utilization ratios of *A. niger* 20-15 and 21-8 were respectively improved by 44.7% and 26.3% compared to *A. niger* CGMCC 10142. What’s more, we found that the reducing sugar concentrations increased rapidly during the first 16 h, and especially during the first 8 h. It then decreased in the subsequent fermentation period with increasing CA production, during which a large amount of glucose was utilized to synthesize CA. Consequently, the reducing sugar concentration decreased rapidly after 24 h. Therefore, the HGT1-overexpressing transformants exhibited a higher glucose utilization ability which was also reflected in an increase of CA production.

We also calculated the glucose-CA conversion rate based on the measured values of total sugar consumption and CA production, as shown in Fig. 5C. As expected, the HGT1-overexpressing strains showed higher conversion rates than the original strain. The final conversion rates of *A. niger* 20-15 and 21-8 were 102.4 and 100.4%, respectively. The CA fermentation performance was in the order *A. niger* 20-15 > *A. niger* 21-8 > *A. niger* CGMCC 10142.

### Transcription levels of HGT1, citrate synthase and glucokinase genes in recombinant *A. niger*

The relative transcription levels of the *HGT1* gene (XM_001399160.2), *citrate synthase* (CS) gene (XM_001393946), and *glucokinase* gene (XM_001395875.2) (The GenBank access numbers are derived from *A. niger* CBS 513.88) during the fermentation process were measured to explore whether the overexpression of HGT1 influenced the metabolism and accumulation of CA in *A. niger*. Mycelial pellet samples of *A. niger* 20-15 (P*gla*A) and 21-8 (P*aox1*) were taken at 12 and 48 h, separated from the culture supernatant through filtration, and the total RNA was extracted for real-time quantitative PCR.

**Fig. 6:**
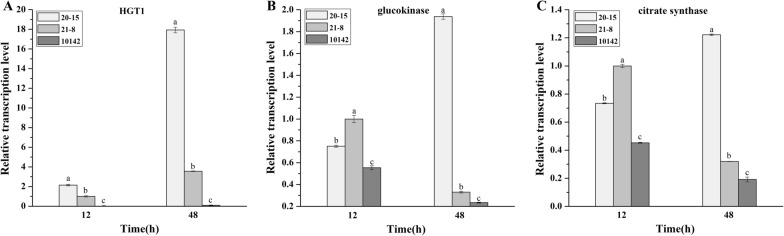
Real-time qPCR analysis of the expression profile of *HGT1*, *glucokinase*, and *citrate synthase* at 12 and 48 h in *A. niger* 20-15, 21-8 and 10142 strain. The *18S rRNA* was used as an internal control. Vertical bars represented the mean ± S.D (n = 3). **A** The relative transcription level of *HGT1* at 12 and 48 h in *A. niger* 20-15, 21-8 and 10142 strain; **B** The relative transcription level of *glucokinase* at 12 h and 48 h in *A. niger* 20-15, 21-8 and 10142 strain; **C** The relative transcription level of *citrate synthase* at 12 and 48 h in *A. niger* 20-15, 21-8 and 10142 strain. The samples at each time-point with different letters differ significantly (abc, P < 0.05)

The relative expression of HGT1 is shown in Fig. 6A. The transcription level of *HGT1* in the overexpression strains was much higher than in the control at 12 and 48 h. The transcription level of *HGT1* in *A. niger* 20-15 was about 219 times and 208 times higher than in the original strain at 12 and 48 h, respectively, which indicated that the glucose utilization efficiency of the HGT1 overexpression strain was also higher than that of *A. niger* CGMCC 10142. Moreover, the HGT1 overexpression strain *A. niger* 20-15 with the P*gla*A promoter showed 2.5 times higher transcription than the strain *A. niger* 21-8 with the P*aox1* promoter at 12 h, as well as 5 times higher at 48 h. P*gla*A is a widely used strong fungal promoter that can be induced by starch or dextrose [37] and P*aox1* is the native promoter of alternative oxidase in *A. niger* [8]. The transformant 20-15 (P*gla*A) with higher HGT1 transcription level didn’t present a significantly more CA yield than 21-8 (P*aox1*) (Fig. 3A), probably because the content of HGT1 protein in the strains 20-15 and 21-8 was almost the same. Besides, all the HGT1 transcription of the strains at 48 h was at least 2 times higher than that of 12 h, indicating HGT1 did not function effectively at high sugar concentrations at the fermentation beginning. The overexpression of the low-affinity glucose transporter increased the substrate uptake at high glucose concentrations, partly overcoming the limitation of glucose consumption in the strains. The HGT1 transporter accelerated sugar transport at the end of CA fermentation when the remaining glucose concentration was low. This could reduce the final total residual sugar concentration and improve the economic value of the fermentation.

Notably, the relative expression levels of glucokinase in the HGT1 overexpression strains were higher than in the parental strain, especially at 48 h (Fig. 6B). The glucokinase expression of *A. niger* 20-15 was 8.4 times higher than in the parental strain, while that of *A. niger* 21-8 was 1.4 times higher at 48 h, which indicated that the overexpression strains had a higher glucose utilization rate and improved CA production ability. These results indicated that the overexpression of HGT1 increased the glucose uptake efficiency, which provided adequate carbon flux for increased CA production in the transformants. Glucokinase plays a key role in the metabolic activation of glucose to glucose 6-phosphate, and its activity is controlled via feedback inhibition by glucose 6-phosphate and ADP. The increased glucokinase activity could therefore directly improve the CA production at the end of the fermentation process.

As shown in Fig. 6C, the expression of citrate synthetase (CS) presented a similar trend to glucokinase. The CS expression of the engineered *A. niger* strain 20-15 was 6.4 times higher than that of the parental strain at 48 h. CS is necessary for the synthesis of CA as indicated by its name and therefore plays a key role in CA metabolism. When the *cs* gene is deleted in *A. niger*, CA production from 98.7 falls to 64.3 g/L [38]. High expression of HGT1, therefore, increased the expression of CS and contributed to abundant CA accumulation. The changes in the relative expression of CS indicated that high carbon flux increased the CA yields of the transformants. It is therefore possible that substrate limitation is one of the reasons for low enzyme activity in the parental strain.

Though some genes such as *aox1* and *glaA* have strong promoters available for *A. niger* engineering, these genes may be also expressed in *A. niger*, which may affect citric acid production [8, 39]. We therefore speculated that P*aox1* may not only drive the expression of the *HGT1* gene but also activate its own *aox1* gene to some extent, and thereby indirectly increase the fermentation performance. As was observed in *A. niger* 21-8, higher transcription levels of *glucokinase* and *CS* than *A. niger* 20-15 at 12 h when the mycelium grows vigorously and dissolved oxygen becomes limited. In view of this, it may be useful to verify the relative expression of *aox1* and related enzymes in future studies. What’s more, the exact insertion locus or copy numbers of the overexpression locus may also influence the expression levels. The enzymes that are important for CA synthesis, such as CS and glucokinase were expressed at higher levels in the HGT1 overexpression strains than in *A. niger* CGMCC 10142. It is possible that sufficient glucose increased the expression of these enzymes and thereby improved the CA production performance of *A. niger* [20]. Furthermore, the starting strain used in this study is already an industrial CA production strain, and its metabolite levels are much higher than in undomesticated wild-type strains.

Glucose transport is the first step from sugar to CA, but it does not influence the CA fermentation directly. In this study, we found that the overexpression of HGT1 improved the CA fermentation performance in terms of the total consumption of reducing sugar and total residual sugar remaining in the fermentation broth. Furthermore, the final glucose-CA conversion rates of *A. niger* 20-15 and 21-8 both increased to 102.4% and 100.4%, which was close to the theoretical glucose-CA conversion rate of 106.6% (As depicted in the chemical reaction formula from glucose to citric acid: C_6_H_12_O_6_ (MW: 180.15) + 1.5O_2_ → C_6_H_8_O_7_ (MW: 192.12) + 2H_2_O) when all the resources are used to produce CA and none for growth [22]. Finally, the CA production of the engineered strains also increased by 7.3% for *A. niger* 20-15 and 4.4% for *A. niger* 21-8. Therefore, sugar intake may still be a bottleneck in the fermentation process.

## Conclusions

In this study, we investigated the effects of overexpressing the glucose transporter HGT1 on industrial CA fermentation and found that it is beneficial for CA production in both shake flasks and a 30 L laboratory-scale bioreactor. The increase in CA production can be explained by an increased sugar utilization rate and indirect positive effects on the expression of key metabolic genes. Overexpression of glucose transporter genes can be used to reduce the cost of industrial-scale production and increase the yield of citric acid.

## Material and methods

### Strains, reagents, and culture conditions

All the strains and plasmids used in this work were obtained from the Tianjin Key Laboratory of Industrial Fermentation Microbiology, Tianjin, China, or constructed in this work, as shown in Table 1. The parental strain *A. niger* CGMCC 10142 was cultivated on potato dextrose agar (PDA) at 35 ℃. *Escherichia coli* DH5α was cultured in LB medium at 37 ℃ for plasmid propagation. *Agrobacterium tumefaciens* AGL1 was cultured in LB medium at 28 ℃ for plasmid transformation. All the reagents were analytically pure. *Hygromycin* was purchased from Solarbio. Co., Ltd (China). The reagents for plasmid construction and real-time qPCR were bought from Takara Co., Ltd (China).

**Table 1:** Strains and plasmids used in this work

Name	Genetic characteristics	From
*Aspergillus niger* CGMCC 10142	CA producer, parental strain	The laboratory
*Aspergillus niger* 20-15	P*gla*A induced *HGT1* overexpression strain, *hyg*^r^	This work
*Aspergillus niger* 21-8	P*aox1* induced *HGT1* overexpression strain, *hyg*^r^	This work
*E. coli* DH5α	plasmid propagation	The laboratory
*A. tumefaciens* AGL1	plasmid transformation	The laboratory
pGM-*HGT1*	T-vector with *A. niger HGT1* DNA, *Amp*^r^	This work
p80-HSVtk	*ku70* gene knockout plasmid, *hyg*^r^ *Kan*^r^	The laboratory
p20	*HGT1* overexpression plasmid with promoter P*gla*A, *hyg*^r^ *Kan*^r^	This work
p21	HGT1 overexpression plasmid with promoter P*aox1*, *hy*g^r^ *Kan*^r^	This work

Spores of *A. niger* CGMCC 10142 or its transformants were obtained from cultures on PDA plates which were grown at 35 °C for 4 days, and cultured in corn steep liquor (RZBC Co., Ltd., Rizhao, Shandong, China) with a concentration up to 1 × 10^5^ spores/mL. *A. niger* was cultured at 35 ℃ for 72 h under constant shaking at 330 r/min in 500 mL shake flasks containing 50 mL of medium. The amount of 1 × 10^5^ spores/mL was inoculated in 30 L fermenters containing 20 L corn steep liquor, and cultivated at 0.1 MPa, 330 L/h aeration rate, 350 r/min, and 35 °C for 64 h, according to the previous report [39].

### Construction of *HGT1* gene overexpression plasmids and strains

All primers used for plasmid construction are listed in Table 2. The *HGT1* gene, *gla*A promoter (P*gla*A), and *aox1* promoter (P*aox1*) were cloned from the genome of *A. niger* CGMCC 10142. The genomic DNA was extracted from young hyphae grown on PDA medium. The *A. niger* CBS 513.88 genome was used as a reference to design primers. After sequencing at BGI Genomics Co., Ltd (China), we conducted sequence alignment based on NCBI to investigated the coherence of the HGT1 sequence. The plasmid p80-HSVtk which contains HSVtk, *ku70* upstream sequence, *hygromycin* resistance marker (*hyg*), and *ku70* downstream sequence was used as the parental plasmid. The *ku70* deletion leads to improved efficiency of transformation and the HSVtk insertion leads to an increased rate of homologous recombination [40]. The p80-HSVtk plasmid was cut with *Kpn* I after which promoter and HGT1 coding sequence were ligated using the Multis kit (Vazyme Biotech Co., Ltd. China) to form the plasmids p20 and p21 (Additional file 1: Figure S1a, b). The HGT1 coding sequence was expressed using the P*gla*A promoter in the p20, and P*aox1* in p21.

**Table 2:** The primers used in this work

Name	Primer sequence (5´ → 3´)	Amplification product	Length (bp)
*Hyg*-F	GTCGACGTTAACTGATATTG	*hygromycin* resistance gene (*hyg*)	1389
*Hyg*-R	TTTGCCCTCGGACGAGTGCT		
*Ku70*-L-F	GGGGTACCGAGCTCGAGGCCAAACAGGCAG	*ku70*	1800
*Ku70*-R-R	CCCAAGCTT TCTAGATAACTGTACATCGCCT		
P*gla*A-F	CGATAGATCTGGATCCCTGCTCTCTCTCTGCTCT	*gla*A promoter	1493
P*gla*A-R	CAACAACATGTGAGGAGGTGAACGAA		
*HGT1*-F	CACCTCCTCACATGTTGTTGATTGGCAACAT	*HGT1*	1819
*HGT1*-R	CGACTCTAGAGGATCCTTATGCTGTGGCCTCCTGGG		
P*aox1*-F	CGATAGATCTGGATCCGACACCGAGCACATGA	*aox1* promoter	1404
P*aox1*-R	GTTGCCAATCAACATCGGGTATAGAACCACAG		
GKqrt-F	CCGCAATGAGAAGAATGG	qPCR for glucokinase	
GKqrt-R	CATCGGGAATGTTGAAGC		
CSqrt-F	ACGGCAAGACCAAGAACC	qPCR for citrate synthase	
CSqrt-R	CACGGGAAACACCGAAGA		
HGT1qrt-F	CGGTATGCTCGTTGTTGG	qPCR for HGT1	
HGT1qrt-R	GGCGAAAGTTCACTGATGTA		
18Sqrt-F	TCGCTACTACCGATTGAA	qPCR for 18S rRNA	
18Sqrt-R	CACCTACGGAAACCTTGT		

The *EcoR* I, *Kpn* I, *Pst* I and *Hind* III sites are underlined

The plasmids p20 and p21 were individually introduced into *A. niger* CGMCC 10142 by *Agrobacterium*-mediated transformation according to previously reported methods [41, 42]. The cells were cultured on fungal complete medium (CM) with 200 μg/mL *hygromycin* to screen the correct transformants and the CM medium reported by Michielse and colleagues [35].

### Analytical methods for CA and generation of stable strains

The fresh hyphae of transformants from CM + *hygromycin* medium were first transferred to CM + 0.5% CaCO_3_ plates and grown at 35 ℃ for 72 h to check the CA yields of the strains via comparing the ratios of the transparent halo zones to the colony diameters. A larger acid-etched cycle indicates a greater acid production potential. Then, the transformants obtained that showed large transparent halo zones were verified by PCR amplification. After verifying the inserted segments, spore suspensions of the correct transformants were prepared for the shake flask fermentations.

Selected transformants were subcultured on CM medium for 15 generations, and then the genomic DNA was extracted to verify their genetic stability. Additionally, CA production in shake flask fermentation was tested again.

### Determination of sugar and citric acid yields in the 30 L fermenter

The cell pellets were checked under a microscope every 8 h in the 30 L fermentation process. Total sugar, reducing sugar and CA concentrations were also measured every 8 h. The contents of residual total sugar and residual reducing sugar were determined using the DNS (3,5-dinitrosalicylic acid) method [43]. Briefly, 1 mL of diluted supernatant along with 1 mL of DNS reagent was placed in a boiling water bath for 5 min then cooled to room temperature. Then, the absorbance at 540 nm (A _540_) was measured to determine the reducing sugar. Total sugar in supernatants needs adding equal volumes 6 mol/L HCl and heating for 30 min in a boiling-water bath, adjust the pH to neutral, then were also determined by DNS method. Standard measurements were performed using the same procedure.

Citric acid was measured using a commercially available test kit (Test Kit Cat. No:10139076035, Roche, Germany). Briefly, to 0.2 mL sample, 1 mL solution 1 (containing glycylglycine buffer (pH = 7.8), 11.33 U L-malate dehydrogenase, 23.3 U L-lactate dehydrogenase, 0.42 mg NADH) and 1.8 mL double-distilled water were added. After incubation at 20–25℃ for 5 min, read the A _340_. After that, 20 μl solution 2 (containing 0.8 U citrate lyase) was added for a further 5 min. The change in A _340_ was monitored after the reaction with citrate lyase. Standard measurements were performed using the same procedure.

### Real-time quantitative PCR

To further investigate how the glucose transporter affects the metabolism and accumulation of CA in *A. niger*, real-time quantitative PCR was conducted during the fermentation. The relative expression of *HGT1* and the genes encoding key rate-limiting enzymes related to the citric acid cycle, citrate synthase and glucokinase, were measured. The primers used for the qPCR are listed in Table 2. The pellet of 12 and 48 h in CA fermentation were filtered to extract total RNA. Then, reverse transcription was conducted.

The relative gene transcription levels were calculated using the ${2}^{{ - \Delta \Delta {\text{C}}_{{\text{t}}} }}$ method, and the values were normalized to the 18S rRNA (CGMCC 10142). A standard curve using a dilution series of the cloned amplicon was used to calculate the gene-specific real-time qPCR efficiency. The correlation coefficient (R^2^) of the slope of the standard curve was used to calculate each gene’s PCR amplification efficiency as described before [8].

### Statistical analysis

All the results were based on three independent experiments, and samples with P < 0.05 were considered significant.

## Supplementary Information

**Additional file 1**: **Figure S1**. The recombinant plasmid map of p20 and p21. (a) The recombinant plasmid (p20) of overexpression HGT1 transcripted by glaA promoter (PglaA); (b) The recombinant plasmid (p21) of overexpression HGT1 transcripted by aox1 promoter (Paox1). **Figure S2**. Verification of HGT1 gene overexpression plasmids and strains. (a) Verification of the plasmid p20, M: DNA marker III, 1: hyg (1389 bp), 2: PglaA -HGT1 (3312 bp), 3: HGT1-hyg (3217 bp), 4: PglaA–hyg (4710 bp); (b) Verification of the plasmid p21, M: DNA marker III, 1: Paox1-hyg (4612 bp), 2: HGT1-hyg (3217 bp), 3: hyg (1389 bp), 4: Paox1-HGT1 (3223 bp); (c) Verification of A. tumefaciens p20 and p21, M: DNA marker III, 1: p20 plasmid control, 2-3: A. tumefaciens p20 verifying of PglaA-HGT1 (3312 bp), 4: p21 plasmid control, 5-6: A. tumefaciens p21 verifying of Paox1-HGT1 (3217 bp); (d) Verification of A. niger p20 transformants, M: DNA marker III, 1: p20 plasmid control, 2: negative control, 3-10: p20 transformants genome verifying of PglaA-HGT1 (3312 bp); (e) Verification of A. niger p21 transformants M: DNA marker III, 1: p21 plasmid control, 2: negative control, 3-10: p21 transformants genome verifying of Paox1-HGT1(3217 bp); (f) HTG1-hyg verifation M: DNA marker III 1: plasmid control, 2: negative control, 2-10: PCR product of HTG1-hyg (3217 bp) from transformants genome; (g) Verifying from ku70 upstream to downstream of the transformants M: DNA marker III 1: plasmid control (5710 bp), 2 negative control (1800 bp), 3-10: transformants genome ku70 amplification. **Figure S3**. Multiple sequence alignment of glucose transporters. **Figure S4**. Validating hyg gene in the HGT1 overexpression mutants at the 15th generation. M: DNA marker III, 1: positive control (1389 bp), 2: negative control, 3-10: amplified hyg gene using the genomic DNA of overexpressed mutants. Genomes of all strains were gained from a 15-generation subculture. **Figure S5**. Measurement of diameter size of mycelial balls of citric acid in the 30 L bioreactor fermentation.

**Additional file 2**: **Table S1**. The initial screening of high-yield citric acid transformants.

## Data Availability

The authors declare that the resequencing results of *A. niger* CGMCC 10142 genome cannot be made public for the time being, because the study on the genome has not been published and the international patent is under application.

## Abbreviations

HGT1: High-affinity glucose transporter 1
CS: Citrate synthase
CA: Citrate acid
DNS: 3,5-Dinitrosalicylic acid
