# Supplementary material for: Improving citric acid production of an industrial Aspergillus niger CGMCC 10142: identification and overexpression of a high-affinity glucose transporter with different promoters
Source: Microb Cell Fact. 2021 Aug 26;20:168. doi: 10.1186/s12934-021-01659-3 (PMC8394697; doi:10.1186/s12934-021-01659-3)
Supplement: Supplementary file 2 — Additional file 2: Table S1. The initial screening of high-yield citric acid transformants. [file 12934_2021_1659_MOESM2_ESM.docx]

Table S1 The initial screening of high-yield citric acid transformants

| Strains | Colony diameter (mm) | Acid ring diameter (mm) | Ring diameter ratio | Strains | Colony diameter (mm) | Acid ring diameter (mm) | Ring diameter ratio |
| --- | --- | --- | --- | --- | --- | --- | --- |
| p20-1 | 8.0 | 24.0 | 3.0 | p21-1 | 7.5 | 24.0 | 3.2 |
| p20-2 | 8.0 | 28.0 | 3.5 | p21-2 | 7.0 | 19.6 | 2.8 |
| p20-3 | 12.0 | 32.0 | 2.7 | p21-3 | 12.0 | 37.0 | 3.1 |
| p20-4 | 7.5 | 25.0 | 3.3 | p21-4 | 10.0 | 27.0 | 2.7 |
| p20-5 | 7.5 | 22.0 | 2.9 | p21-5 | 3.0 | 8.0 | 2.8 |
| **p20-15** | **10.0** | **39.0** | **3.9** | **p 21-8** | **11.0** | **42.0** | **3.8** |
| **p20-16** | **10.0** | **38.0** | **3.8** | p21-9 | 11.0 | 29.0 | 2.6 |
| p20-17 | 6.0 | 21.0 | 3.5 | p21-10 | 12.5 | 36.0 | 2.9 |
| p20-18 | 7.0 | 22.0 | 3.1 | p21-11 | 10.5 | 34.0 | 3.2 |
| p20-19 | 3.5 | 12.0 | 3.4 | p21-12 | 6.5 | 18.0 | 2.8 |
| p20-20 | 6.5 | 33 | 5.0 | p21-13 | 7.5 | 21.0 | 2.8 |
| p20-21 | 4.5 | 20.0 | 4.4 | p21-14 | 8.0 | 26.0 | 3.3 |
| p20-22 | 5.0 | 16.0 | 3.2 | p21-15 | 7.0 | 21.0 | 3.0 |
| p20-23 | 7.0 | 21.0 | 3.0 | p21-16 | 11.0 | 34.0 | 3.1 |
| p20-24 | 10.0 | 30.0 | 3.0 | p21-17 | 12.0 | 31.0 | 2.6 |
| **p20-25** | **8.5** | **32.0** | **3.8** | p21-18 | 9.5 | 30.0 | 3.2 |
| p20-26 | 13.0 | 45.0 | 3.5 | p21-19 | 8.0 | 19.0 | 2.4 |
| **p20-27** | **12.0** | **44.0** | **3.7** | p21-20 | 8.5 | 27.0 | 3.2 |
| p20-28 | 8.0 | 43 | 5.3 | p21-21 | 9.5 | 28.0 | 2.9 |
| **p20-29** | **11.0** | **43.0** | **3.9** | p21-22 | 10.0 | 31.0 | 3.1 |
| p20-30 | 12.0 | 41.0 | 3.4 | p21-23 | 11.5 | 37.0 | 3.2 |
| p20-31 | 18.0 | 6.0 | 3.0 | p21-24 | 8.0 | 21.0 | 2.6 |
| p20-32 | 13.0 | 43.0 | 3.3 | p21-25 | 4.0 | 11.0 | 2.7 |
| p20-33 | 12.0 | 41.0 | 3.4 | p21-26 | 3.0 | 7.5 | 2.5 |
| p20-34 | 15 | 36.0 | 2.4 | p21-27 | 9.0 | 28.0 | 3.1 |
| p20-35 | 15.0 | 43.0 | 2.8 | p21**-28** | **11.0** | **39.5** | **3.6** |
| p20-36 | 13.0 | 38.0 | 2.9 | p21-29 | 13.0 | 42.0 | 3.2 |
| p20-37 | 13.0 | 42.0 | 3.2 | p21-30 | 4.5 | 11.0 | 2.4 |
| p20-38 | 18.5 | 37.5 | 2.0 | p21-31 | 10.0 | 25.0 | 2.5 |
| p20-39 | 9.0 | 27.8 | 3.1 | p21**-32** | **12.5** | **46.0** | **3.7** |
| p20-40 | 11.0 | 36.4 | 3.3 | p21-33 | 12.0 | 36.0 | 3.0 |
| p20-41 | 13.0 | 38.0 | 2.9 | p21-34 | 11.0 | 30.0 | 2.7 |
| p20-42 | 12.0 | 41.0 | 3.4 | p21-35 | 11.0 | 31.0 | 2.8 |
| p20-43 | 13.0 | 42.0 | 3.2 | p21-36 | 12.5 | 32.5 | 2.6 |
