# Supplementary material for: Improving citric acid production of an industrial Aspergillus niger CGMCC 10142: identification and overexpression of a high-affinity glucose transporter with different promoters
Source: Microb Cell Fact. 2021 Aug 26;20:168. doi: 10.1186/s12934-021-01659-3 (PMC8394697; doi:10.1186/s12934-021-01659-3)
Supplement: Supplementary file 1 — Additional file 1: Figure S1. The recombinant plasmid map of p20 and p21. (a) The recombinant plasmid (p20) of overexpression HGT1 transcripted by glaA promoter (PglaA); (b) The recombinant plasmid (p21) of overexpression HGT1 transcripted by aox1 promoter (Paox1). Figure S2. Verification of HGT1 gene overexpression plasmids and strains. (a) Verification of the plasmid p20, M: DNA marker III, 1: hyg (1389 bp), 2: PglaA -HGT1 (3312 bp), 3: HGT1-hyg (3217 bp), 4: PglaA–hyg (4710 bp); (b) Verification of the plasmid p21, M: DNA marker III, 1: Paox1-hyg (4612 bp), 2: HGT1-hyg (3217 bp), 3: hyg (1389 bp), 4: Paox1-HGT1 (3223 bp); (c) Verification of A. tumefaciens p20 and p21, M: DNA marker III, 1: p20 plasmid control, 2-3: A. tumefaciens p20 verifying of PglaA-HGT1 (3312 bp), 4: p21 plasmid control, 5-6: A. tumefaciens p21 verifying of Paox1-HGT1 (3217 bp); (d) Verification of A. niger p20 transformants, M: DNA marker III, 1: p20 plasmid control, 2: negative control, 3-10: p20 transformants genome verifying of PglaA-HGT1 (3312 bp); (e) Verification of A. niger p21 transformants M: DNA marker III, 1: p21 plasmid control, 2: negative control, 3-10: p21 transformants genome verifying of Paox1-HGT1(3217 bp); (f) HTG1-hyg verifation M: DNA marker III 1: plasmid control, 2: negative control, 2-10: PCR product of HTG1-hyg (3217 bp) from transformants genome; (g) Verifying from ku70 upstream to downstream of the transformants M: DNA marker III 1: plasmid control (5710 bp), 2 negative control (1800 bp), 3-10: transformants genome ku70 amplification. Figure S3. Multiple sequence alignment of glucose transporters. Figure S4. Validating hyg gene in the HGT1 overexpression mutants at the 15th generation. M: DNA marker III, 1: positive control (1389 bp), 2: negative control, 3-10: amplified hyg gene using the genomic DNA of overexpressed mutants. Genomes of all strains were gained from a 15-generation subculture. Figure S5. Measurement of diameter size of mycelial balls of citric aci [file 12934_2021_1659_MOESM1_ESM.docx]

a	b

Figure S1. The recombinant plasmid map of p20 and p21. (a) The recombinant plasmid (p20) of overexpression HGT1 transcripted by *gla*A promoter (P*gla*A); (b) The recombinant plasmid (p21) of overexpression HGT1 transcripted by *aox1* promoter (P*aox1*).

Figure S2. Verification of *HGT1* gene overexpression plasmids and strains. (a) Verification of the plasmid p20, M: DNA marker Ⅲ, 1: *hyg* (1389 bp), 2: P*gla*A -HGT1 (3312 bp), 3: HGT1-*hyg* (3217 bp), 4: P*gla*A–*hyg* (4710 bp); (b) Verification of the plasmid p21, M: DNA marker Ⅲ, 1: P*aox1*-*hyg* (4612 bp), 2: HGT1-*hyg* (3217 bp), 3: *hyg* (1389 bp), 4: P*aox1*-HGT1 (3223 bp); (c) Verification of *A. tumefaciens* p20 and p21, M: DNA marker Ⅲ, 1: p20 plasmid control, 2-3: *A. tumefaciens* p20 verifying of P*gla*A-*HGT1* (3312 bp), 4: p21 plasmid control, 5-6: *A. tumefaciens* p21 verifying of P*aox1*-*HGT1* (3217 bp); (d) Verification of *A. niger* p20 transformants, M: DNA marker Ⅲ, 1: p20 plasmid control, 2: negative control, 3-10: p20 transformants genome verifying of P*gla*A-*HGT1* (3312 bp); (e) Verification of *A. niger* p21 transformants M: DNA marker Ⅲ, 1: p21 plasmid control, 2: negative control, 3-10: p21 transformants genome verifying of P*aox1*-*HGT1*(3217 bp); (f) *HTG1*-*hyg* verifation M: DNA marker Ⅲ 1: plasmid control, 2: negative control, 2-10: PCR product of *HTG1*-*hyg* (3217 bp) from transformants genome; (g) Verifying from *ku70* upstream to downstream of the transformants M: DNA marker Ⅲ 1: plasmid control (5710 bp), 2 negative control (1800 bp), 3-10: transformants genome *ku70* amplification.

Figure S3. Multiple sequence alignment of glucose transporters.

Figure S4. Validating *hyg* gene in the HGT1 overexpression mutants at the 15th generation. M: DNA marker Ⅲ, 1: positive control (1389 bp), 2: negative control, 3-10: amplified *hyg* gene using the genomic DNA of overexpressed mutants. Genomes of all strains were gained from a 15-generation subculture.

Figure S5. Measurement of diameter size of mycelial balls of citric acid in the 30 L bioreactor fermentation.
